# Reciprocal control of lncRNA-BCAT1 and β-catenin pathway reveals lncRNA-BCAT1 long non-coding RNA acts as a tumor suppressor in colorectal cancer

**DOI:** 10.18632/oncotarget.15466

**Published:** 2017-02-17

**Authors:** Fei Xie, Xudong Xiang, Qionglin Huang, Pengzhan Ran, Yuncang Yuan, Qian Li, Guoxiang Qi, Xiaopeng Guo, Chunjie Xiao, Shangyong Zheng

**Affiliations:** ^1^ School of Medicine, Yunnan University, Kunming, Yunnan 650091, P.R. China; ^2^ Department of Thoracic Surgery, The Third Affiliated Hospital of Kunming Medical University, Kunming, Yunnan 650118, P.R. China

**Keywords:** long non-coding RNA, lncRNA-BCAT1, β-catenin, tumor suppressor, colorectal cancer

## Abstract

β-catenin plays a major role in tumor development and progression. The present study found that β-catenin was upregulated in 30 samples of colorectal cancer (CRC) tissue as compared to adjacent non-tumor tissues. Analysis of long non-coding RNA (lncRNA) expression profiles using the GSE18560 and GSE44097 datasets, which were generated via the Affymetrix plus 2.0 microarray platform and downloaded from the GEO database, revealed 20 differentially expressed lncRNAs following β-catenin knockdown. We focused on AK091631, a novel lncRNA, which we named lncRNA-β-catenin associated transcript 1 (LncRNA-BCAT1). lncRNA-BCAT1 expression was decreased in CRC tissues, and was negatively associated with β-catenin in both CRC tissues and cell lines. lncRNA-BCAT1 overexpression suppressed CRC cell growth and invasion by downregulating cyclin D1, c-Myc, and MMP-2. These results suggest that lncRNA-BCAT1 overexpression inhibits CRC cell growth and invasion via Wnt/β-catenin pathway blockade, and that lncRNA-BCAT1 is repressed by Wnt/β-catenin signaling. This evidence suggests that lncRNA-BCAT1 is a tumor suppressor and that lncRNA-BCAT1 may be an effective prognostic biomarker in CRC.

## INTRODUCTION

Colorectal cancer (CRC) is the fourth most common cancer and the third leading cause of cancer death worldwide, resulting in more than 500,000 deaths and composing 10% of new cancer diagnoses annually [1–3]. More than 10% of CRC patients are diagnosed with advanced stage disease, and about 30% of patients diagnosed with early stage CRC will develop metastatic disease [4]. The 5-year survival rate decreases drastically with advanced stages: 90% for local tumors in initial stages and 12% in advanced stages with metastasis. Because clinical symptoms are often not identified until later stages, late diagnoses lead to reduced patient survival rates [5, 6]. Still, increasing knowledge of the CRC genomic landscape, and targeted agent refinements have improved CRC treatment outcomes [7, 8]. Clinical trials are now being designed to assess patient-specific therapies based on individual patient genotyping and expression profiling. Various lncRNAs are reportedly associated with CRC tumorigenesis and progression. Refining our understanding of relationships between lncRNAs and downstream signaling pathways in CRC could inform novel treatment strategies.

lncRNAs are endogenous RNAs larger than 200 bases, and some may be greater than 100 kb in length [9, 10]. lncRNAs lack open reading frames and account for 80% of the transcriptome. They are expressed across all mammalian genomes and are major regulators of embryonic pluripotency [11], differentiation, and body axis patterning, promoting developmental transitions and regulating histone modifications, and thus influencing transcriptome epigenetic programs [12]. Many lncRNAs are aberrantly expressed in breast [13], lung [14], and esophageal carcinomas [15]. lncRNAs may regulate cancer development by sustaining tumor cell proliferation, evading growth suppressors, enabling replicative immortality, stimulating angiogenesis, and promoting invasion and metastasis. Cancer cells evade growth suppression by inhibiting expression, activation or function of tumor suppressors such as P53, PTEN, and cyclin-dependent kinase inhibitors [16]. Some lncRNAs are reportedly tumor suppressors, promoting gene silencing and apoptosis.

AK091631, located on chromosome 5, is transcribed as a 2.392-kb lncRNA. We named this lncRNA gene, lncRNA-BCAT1 (β-catenin associated transcript 1), according to the Human Gene Nomenclature Committee (HGNC) guidelines (HUGO gene nomenclature database, 2006 updates). We found no repetitive naming in Genbank and EMBO datasets via BLAST. This report is the first direct investigation of AK091631, which hereafter we refer to as lncRNA-BCAT1.

β-catenin plays key roles in both development and carcinogenesis. Samuels, *et al*. reported that high Wnt/β-catenin pathway member expression is a common CRC feature [17]. Wnt/β-catenin signaling is an effective target for chemotherapy and chemoprevention in CRC [18–21]. β-catenin may also act as a transcription factor in concert with LEF1 and TCF1 to activate downstream target genes [22–24]. This activation induced abnormal hepatocyte proliferation and cell cycle progression to promote tumorigenesis [9, 25–27]. Recent studies suggest have established at least two modes of β-catenin activation in cancer. First, β-catenin is degraded via phosphorylation by GSK3 in the Wnt pathway. Binding to the ligand, Wnt, and surface receptors, Frizzled and LRP-5/6, inhibits GSK3 activity, preventing β-catenin degradation in stem cell renewal [28]. Second, many cancer cells express β-catenin mutated in the N-terminal degradation motif [29]. These mutations may aberrantly activate Wnt signaling, resulting in uncontrolled cell differentiation, and ultimately CRC formation [30–33]. Evidence suggests that β-catenin is mutated in more than 90% of CRCs [34, 35].

This study assessed the relationship between lncRNA-BCAT1 and β-catenin expression in CRC and adjacent non-tumor tissues, and six CRC cell lines. We also investigated the effects of lncRNA-BCAT1 overexpression on Wnt/β-catenin pathway downstream targets, such as cyclin D1, c-Myc, and MMP-2. Our results revealed that lncRNA-BCAT1 overexpression represses CRC cell growth and invasion via Wnt/β-catenin pathway blockade.

## RESULTS

### β-catenin is upregulated in CRC tissues

**Figure 1:**
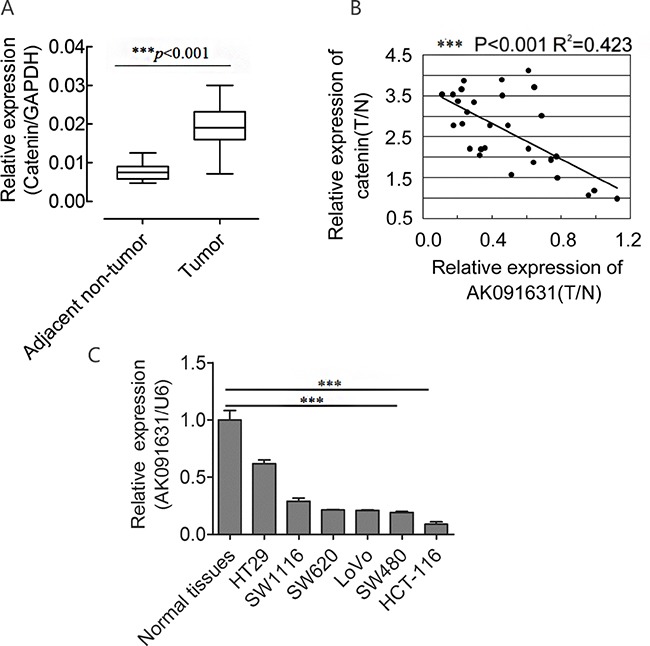
β-catenin up-regulates and is negatively associated with AK091631 (lncRNA-BCAT1) mRNA expression in CRC samples and 6CRC cells Notes: **A**. QRT–PCR analysis of β-catenin expression in the CRC tissues and their corresponding adjacent non-tumor tissues as indicated in Figure 2. The expression of β-catenin was normalized to GAPDH. **B**. A negative Spearman correlation between lncRNA-BCAT1 and β-catenin mRNA levels was found in 30 CRC samples. T, tumor tissues; N, adjacent non-tumor tissues. **C**. QRT–PCR analysis of lncRNA-BCAT1 expression in normal tissues and 6 CRC cells. The expression of lncRNA-BCAT1 was normalized to U6. *p<0.05, **p<0.01, ***p<0.001.

To identify the expression level of β-catenin in CRC, qRT-PCR was conducted to assess β-catenin expression in the 30 CRC and adjacent non-tumor tissues, and normalized to GAPDH. We observed that β-catenin was increased in CRC compared with corresponding adjacent non-tumor tissues (*P*<0.001, Figure 1A), which was in agreement with previous studies [36, 37].

### Differentially expressed lncRNAs in CRC cells after β-catenin knockdown

**Figure 2:**
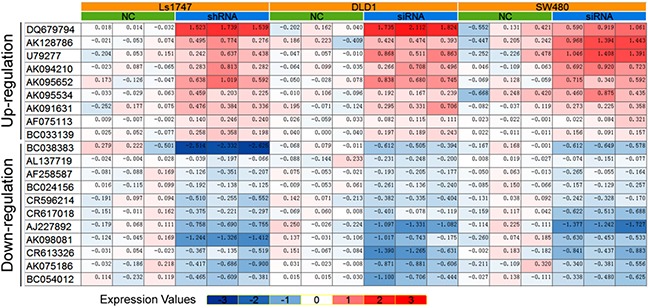
20 LncRNAs were identified consistently in 3 CRC lines after β-catenin knockdown

shRNA- or siRNA-mediated β-catenin knockdown datasets (GSE18560 and GSE44097) generated using the Affymetrix plus 2.0 microarray platform were obtained from GEO for lncRNA expression profile analyses. Expression profiling showed that β-catenin knockdown upregulated lncRNAs, AK091631 (lncRNA-BCAT1), DQ679794, AK128786, U79277, AK094210, AK095652, AK095534, AF075113, and BC033139, and downregulated BC38383, AL137719, AF258587, BC024156, CR596214, CR617018, AJ227892, AK09081, CR613326, AK075186, and BC054012. These lncRNAs expressions were assessed in Ls1747, DLD1 and SW480 CRC cells following β-catenin knockdown (Figure 2).

### lncRNA-BCAT1 verification in CRC tissues

**Figure 3:**
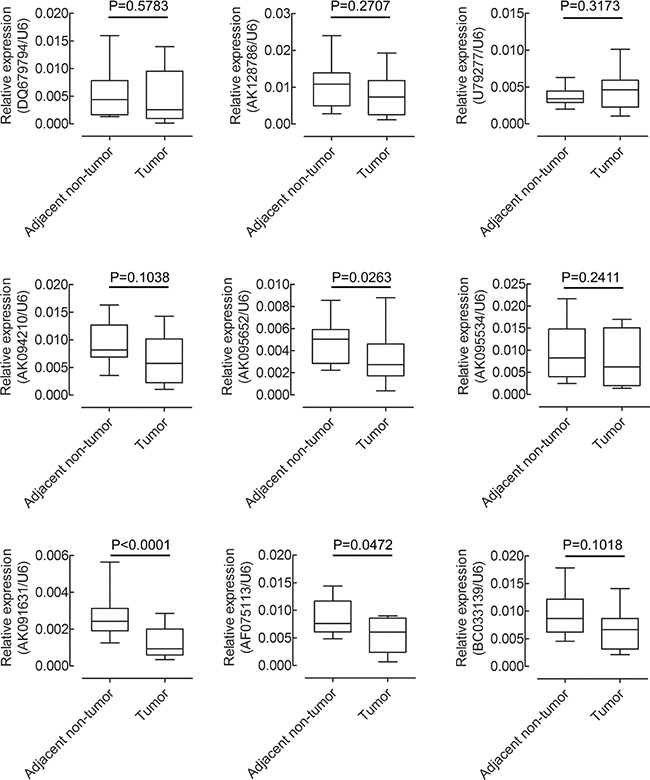
QRT–PCR analysis of 9 up-regulated lncRNAs expression in 30 pairs of CRC tissues and their corresponding adjacent non-tumor tissues The expression of lncRNAs was normalized to GAPDH.

To identify β-catenin-associatedlncRNAs involved in CRC progression, nine lncRNAs upregulated following β-catenin knockdown were selected for further studies. Levels of the selected lncRNAs were detected in 30 CRC and adjacent non-tumor tissues by qRT-PCR and normalized to U6. AK091631 (lncRNA-BCAT1) expression was decreased in CRC tissues compared to corresponding adjacent non-tumor tissues (*P*<0.001, Figure 3). These findings suggested that lncRNA-BCAT1 downregulation might stimulate CRC progression and development.

### lncRNA-BCAT1 is negatively associated with β-catenin in CRC tissues and is downregulated in CRC cell lines

We explored the relationship between lncRNA-BCAT1 and β-catenin expression in CRC tissues. After normalization to adjacent non-tumor tissues, lncRNA-BCAT1 RNA and β-catenin mRNA levels in CRC tissues were analyzed by Pearson's Correlation Coefficient Analysis. lncRNA-BCAT1 was negatively correlated with β-catenin expression in CRC tissues (R^2^=0.423, *P*<0.001) (Figure 1B).

We also analyzed lncRNA-BCAT1 expression via qRT-PCR in six CRC cell lines and normal colorectal tissues. lncRNA-BCAT1 was downregulated in all six lines, especially in HCT-116 and SW480 cells (Figure 1C). Taken together, our results showed that lncRNA-BCAT1 is negatively associated with β-catenin levels in CRC tissues and is down-regulated greatly in the HCT-116 and SW480 cells.

### Effect of lncRNA-BCAT1 on CRC cell proliferation

**Figure 4:**
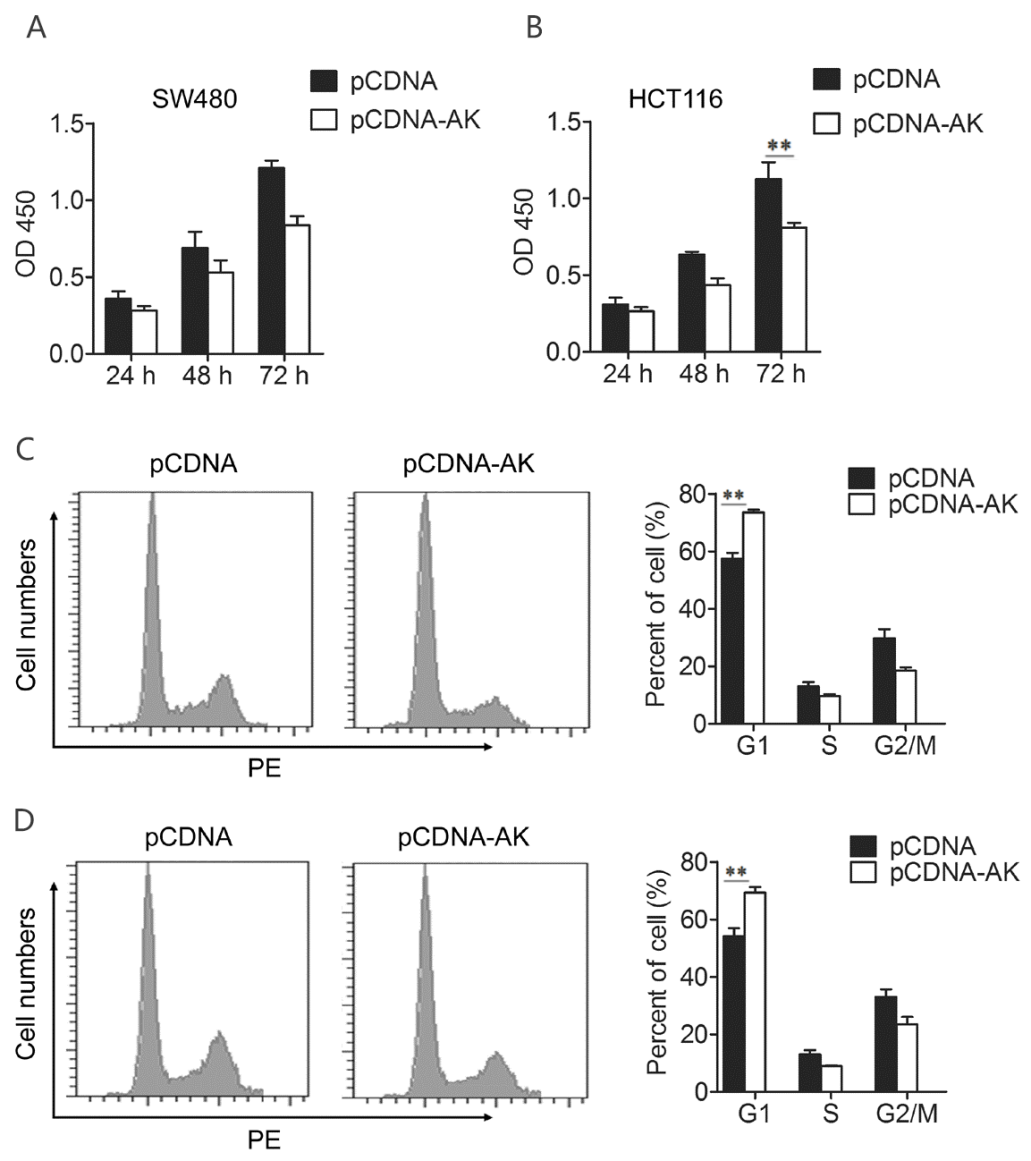
Up-regulation of lncRNA-BCAT1 inhibits the viability of CRC cells Notes: **A, B**. lncRNA-BCAT1 inhibits CRC cells proliferation. SW480 (A) and HCT-116 (B) cells were transfected with pCDNA or pCDNA-BCAT1, and cells proliferation was measured by CCK-8 analysis at 24 h, 48 h, and 72 h after transfection. **C, D**. Cell-cycle distribution was analyzed by FACS analysis. SW480 (C) and HCT-116 (D) cells were transfected with pCDNA or pCDNA-BCAT1. The data were subjected to Student's *t*-test. *p<0.05, **p<0.01, ***p<0.001. AK, AK091631.

To study the effects of lncRNA-BCAT1 on CRC cell proliferation, we performed CCK-8 and cell cycle distribution assays. HCT-116 and SW480 CRC cell lines exhibit low endogenous lncRNA-BCAT1 expression, as confirmed by qRT-PCR (Figure 4C). We transfected the lncRNA-BCAT1 overexpression vector or a control vector into both cell lines. CCK-8 assays revealed that lncRNA-BCAT1 overexpression in HCT-116 and SW480 cells decreased cell proliferation rates (Figure 4A–4B). Cell cycle analysis results showed that cells were arrested in G1 phase (Figure 4C–4D). These results demonstrated that lncRNA-BCAT1 overexpression inhibits CRC cell growth *in vitro*.

### Effect of lncRNA-BCAT1 on CRC cell invasion

**Figure 5:**
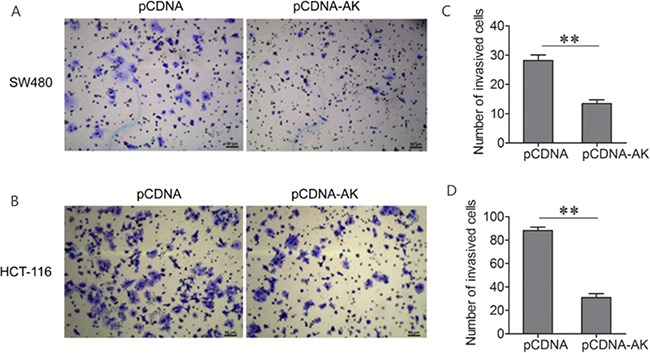
Up-regulation of lncRNA-BCAT1 inhibits the invasion of CRC cells Notes: **A, B**. Transwell invasion assay was performed to detect the invasive properties in SW480 (A) and HCT-116 (B) cells after treated with pCDNA or pCDNA-BCAT1. The data were subjected to Student's *t*-test. *p<0.05, **p<0.01, ***p<0.001. AK, AK091631.

Cell invasion is a significant aspect of cancer progression and metastasis. To investigate whether lncRNA-BCAT1 has a direct functional role in facilitating CRC cell invasion, we evaluated CRC cell invasion using transwell invasion assay. The number of invaded SW480 and HCT-116 cells was reduced after transfection with lncRNA-BCAT1 overexpression vector as compared with the control (Figure 5A–5D). These results showed that the over-expression of lncRNA-BCAT1 inhibits the invasion of CRC cells.

### lncRNA-BCAT1 overexpression inhibits Wnt/β-catenin signaling

Above results showed that lncRNA-BCAT1 is negatively associated with β-catenin mRNA levels in CRC tissues and the over-expression of lncRNA-BCAT1 inhibits the growth and the invasion of CRC cell lines. But its molecular mechanisms remained unclear.

**Figure 6:**
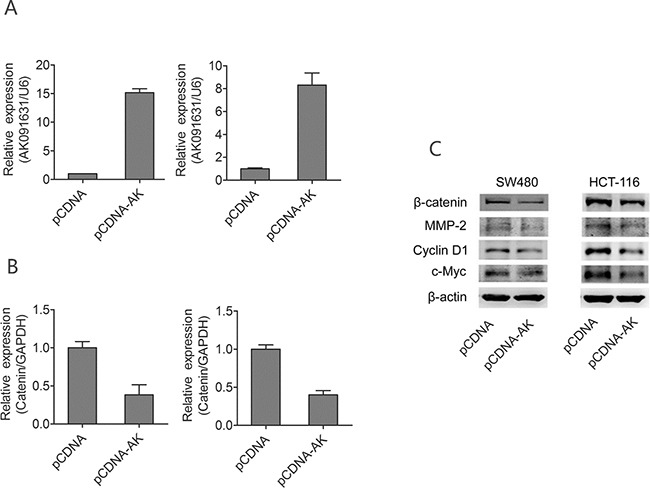
Up-regulation of lncRNA-BCAT1 decrease expression of β-catenin signaling pathway in CRC cells Notes: **A**. SW480 and HCT-116 cells were transfected with pCDNA or pCDNA-BCAT1; 48 h later, the expression of BCAT1 were analyzed qRT–PCR analysis. **B**. SW480 and HCT-116 cells were transfected with pCDNA or pCDNA-BCAT1; 48 h later, the expression of β-catenin were analyzed qRT–PCR analysis. **C**. SW480 and HCT-116 cells were transfected with pCDNA or pCDNA-BCAT1, 48 h later, the expression levels of β-catenin, c-Myc, Cyclin D1, and MMP-2 were analyzed by Western blotting. The data were subjected to Student's *t*-test. *p<0.05, **p<0.01, ***p<0.001. AK, AK091631.

To investigate the molecular mechanisms by which lncRNA-BCAT1 stimulates CRC cell line growth and invasion, we investigated the effects of lncRNA-BCAT1 on Wnt/β-catenin pathway downstream targets in CRC cell line, such as cyclin D1, c-Myc, and MMP-2. lncRNA-BCAT1 expression was upregulated significantly by the lncRNA-BCAT1 overexpression (Figure 6A). While the lncRNA-BCAT1 overexpression downregulated β-catenin expression in CRC cells (Figure 6B), herein the relationship between lncRNA-BCAT1 and β-catenin expression is CRC cell lines is coincidence with their relationship in CRC tissue samples. Based on above results, similarly, β-catenin protein and its downstream targets, cyclin D1, c-Myc, and MMP-2, were also decreased in lncRNA-BCAT1 overexpressing CRC cells (Figure 6C). These data indicated that lncRNA-BCAT1 overexpression inhibits CRC cell growth and invasion via Wnt/β-catenin pathway blockade.

## DISCUSSION

lncRNAs are important regulators in gene expression and tumor development. Identification and investigation of cancer-associated lncRNAs may provide new prognostic biomarkers and therapeutic strategies for cancer treatment [14]. AK091631 (lncRNA-BCAT1) on chromosome 5 is a 2.392 kb lncRNA. This study is the first to correlate lncRNA-BCAT1 with CRC, and to report on its potential anti-tumor mechanisms of action.

Previous studies showed β-catenin upregulation in many cancers, including CRC [38], and β-catenin overexpression is associated with worse patient outcomes [39]. We found the β-catenin expression in CRC was consistent with previous studies. Abnormal lncRNA expression was also previously reported following β-catenin knockdown. For example, lncRNA AK126698 inhibits Wnt/β-catenin pathway activation in A549 cells through Axin1, β-catenin, and other factors [40]. In the present study, analyses of Affymatrix microarray data produced nine upregulated and 11 downregulated lncRNAs in β-catenin knockdown CRC cells, and lncRNA-BCAT1 was particularly increased.

Although the role of Wnt/β-catenin signaling in oncogenesis is well defined, it remained unclear whether β-catenin-associatedlncRNA-BCAT1 was involved in CRC progression. We found the lncRNA-BCAT1 was negatively correlated with β-catenin expression in CRC tissues, and was downregulated in six CRC cell lines (HCT116, SW480, SW620, LOVO, SW-1116 and HT29), especially HCT-116 and SW480 cells. We selected these two lines for further studies.

lncRNA dysregulation is associated with tumorigenesis and metastasis in multiple cancers. lncRNA GAPLINC reportedly promotes CRC cell invasion via binding to PSF/NONO and stimulating SNAI2 expression [41]. lncRNA ncRAN was previously associated with CRC cell migration and invasion [42]. In the present study cell proliferation and cell cycle analysis results showed that lncRNA-BCAT1 overexpression inhibits SW480 and HCT-116 cell growth compared to controls. Similarly, SW480 and HCT-116 cell invasion was reduced as shown by transwell assays.

Wnt/β-catenin signaling is critical in regulating cell proliferation, invasion, and differentiation by modulating downstream target genes, such as cyclin D1, c-Myc and MMP-2 [43, 44]. Our western blotting results demonstrated that lncRNA-BCAT1 overexpression downregulated β-catenin, cyclin D1, c-Myc, and MMP-2. These data revealed that lncRNA-BCAT1 overexpression represses CRC cell growth and invasion via Wnt/β-catenin pathway blockade.

In conclusion, we propose that upregulated β-catenin might inhibit lncRNA-BCAT1 expression by enhancing Wnt/β-catenin signaling, which may promote CRC development. Further studies are needed to assess lncRNA-BCAT1 targets and their functions in CRC. Our results advanced understanding of Wnt/β-catenin signaling in CRC development and progression, and suggest that the likely tumor suppressor lncRNA-BCAT1 may be an effective prognostic CRC biomarker.

## MATERIALS AND METHODS

### Tissue samples and cell lines

Our study was approved by the Yunnan University School of Medicine Ethics Committee. All patients provided written informed consent. Thirty CRC and paired adjacent non-tumor tissue samples were obtained for RT-PCR analysis from the Department of Gastrointestinal Surgery of the Third Affiliated Hospital of Kunming Medical University (Kunming, China). All tissue samples were snap frozen in liquid nitrogen immediately after surgery and stored in liquid nitrogen until use. Additionally, three normal colorectal tissue samples (no colorectal cancer or polyps) were obtained from healthy patients by colonoscopy. The cell lines, HCT-116, SW480, SW620, LoVo, SW1116 and HT29 used in this study were purchased from American Type Culture Collection (ATCC). SW480 and SW620 were cultured in Leibovitz's L-15 medium (Thermo Fisher Scientific, USA) containing 10% fetal bovine serum (FBS) (Life Technologies, Grand Island, USA). HCT-116 and LoVo were cultured in Ham's F12K medium (Thermo Fisher Scientific, USA) containing 10% FBS (Life Technologies, Grand Island, USA). SW116 and HT-29 cells were cultured in RPMI-1640 medium (Thermo Fisher Scientific, USA) containing 10% FBS (Life Technologies, Grand Island, USA). Cells were maintained at 37°C in a humidified atmosphere with 5% CO_2_.

### lncRNA profile mining from Affymatrix microarray data

Raw data from sets, GSE18560 and GSE44097, generated using the Affymetrix plus 2.0 microarray platform, were downloaded from the GEO database. For the GSE18560 dataset, which was produced using Ls1747 CRC cells, only the shRNA-mediated β-catenin knockdown arrays were used. The GSE44097 dataset used DLD1 and SW480 CRC cells with siRNA-mediated knockdown. Studies that generated the datasets were performed in triplicate. Chip definition files (CDFs) with mapping to lncRNAs were downloaded from the GAT Explorer website [http://bioinfow.dep.usal.es/xgate/principal.php] and installed in R version 3.2.0. lncRNA expression profiles were analyzed from the raw CEL files in R with the lncRNA CDFs and Affy packages. Robust Multichip Average method was used to extract lncRNA expression profiles. lncRNAs differentially expressed as a result of β-catenin knockdown were identified in three CRC cell lines. lncRNAs were characterized as upregulated when their minimum values in the treatment group were higher than their maximum values in the negative control groups. Similarly, lncRNAs were considered downregulated when their minimum values in control groups were higher than their maximum values in the treatment group.

### RNA quantification and qRT-PCR assay

Total RNA was isolated from CRC tissues, adjacent non-tumor tissues, and CRC cell lines using Trizol according to the manufacturer's instructions. Purified mRNAs were detected by qRT-PCR assay. Primers are provided in Supplementary Table 1. GAPDH was used as the internal control for lncRNA and β-catenin expression normalization and quantification. Amplification was performed using a Light Cycler 480II (Roche, Basel, Switzerland) and consisted of denaturation at 95°C for 20 s, then 40 cycles of 95°C for 10 s, 55°C for 10 s, and 72°C for 20 s. Fold change in lncRNA expression was calculated using the 2^−ΔΔCt^ method following normalization to GAPDH.

### Plasmid construction and transfection

To generate pCDNA-lncRNA-BCAT1 constructs, the lncRNA-BCAT1 coding sequence was amplified from normal human cDNA and inserted into the KpnI and XhoI sites of the pCDNA construct. Primers are provided in Table 1. All constructs were verified by direct sequencing. Transfections were performed using INTERFERin reagent (Polyplus-transfection). Final plasmid concentrations were 100 ng/μL.

**Table 1:** All primers used in this study

Name	Primer Sequence
GAPDH F	5′-GGAGCGAGATCCCTCCAAAAT-3′
GAPDH R	5′-GGCTGTTGTCATACTTCTCATGG-3′
BCAT1 F	5′-ACTCAGCCATTACAGCACCT-3′
BCAT1 R	5′-GCATGAGTTGAAGCTGCCTT-3′
DQ679794 F	5′-ACTCCTTCTCCCAGTGAACG-3′
DQ679794 R	5′-TAGGTCCAGGGATTGCTTGG-3′
AK128786 F	5′-AGTAACTCAGTGAAGGCCCA-3′
AK128786 R	5′-TCTTCTCTCCAGGCAGCTTT-3′
U79277 F	5′-TGAGACTGGGCACTTGGAAA-3′
U79277 R	5′-GTCTGGCAAGGTTGGACATC-3′
AK094210 F	5′-GGGGAGTTACACAGACACCA-3′
AK094210 R	5′-TGGGTTAGATGCCAAGCTCA-3′
AK095652 F	5′-TGTCACGGAGATGAACACCA-3′
AK095652 R	5′-CGCTGCTCTCTGACTATCCA-3′
AK095534 F	5′-TTTCACTCCCACCTTGTTGC-3′
AK095534 R	5′-GCTCATTGTTGACTTGTGGGA-3′
AF075113 F	5′-GAGCTTCATTTGAGCCCAGG-3′
AF075113 R	5′-ACGCCAGTAAATGACAACAGG-3′
BC033139 F	5′-ACTAAGCCTCAGTGAGCCAG-3′
BC033139 R	5′-TACCAGTCTCCAGGCATCAC-3′
BCAT1 (Vector) F	5′-CCG CTCGAG TGAGAGGCAATGCCTTTCCC-3′
BCAT1 (Vector) R	5′-CGG GGTACC C GTAAGGCTAAAATGACTTTATTTAT-3′

Abbreviations: F, forward primer; R, reverse primer.

### Cell proliferation and cell cycle analysis

SW480 and HCT-116 cells were transfected with pCDNA-lncRNA-BCAT1 or pCDNA using INTERFERin (Polyplus). Cell proliferation was assessed at 24, 48, 72, and 96 h post-transfection using Cell Counting Kit-8 (Dojindo, Kumamoto, Japan) according to the manufacturer's instructions. Briefly, 1×10^4^ cells/well were seeded onto 96-well plates in a final volume of 100 μl. At the indicated times, 10 μl CCK-8 solution was added to each well and cells were incubated at 37°C for 30 min before detecting the absorbance at 450 nm.

For cell cycle assays, 48 h after SW480 and HCT-116 cells were transfected, culture medium without serum was added for 24 h, followed by 6 h in fresh medium with 10% FBS. Cells were then harvested and fixed in 70% ethanol at 4°C overnight. Cells were stained with propidium iodide (PI) (Biolegend, California, USA) solution at a final concentration of 50 μg/ml, containing 50 μg/ml RNase A. Cell cycles were analyzed by flow cytometry (BD LSRII, San Jose, CA, USA).

### Invasion assays

CRC cell invasion was analyzed using 24-well transwell chambers coated with Matrigel (BD Pharmingen, San Jose, CA, USA). Chambers have upper and lower culture compartments that are separated by polycarbonate membranes with 8-μm pores (Costar, Cambridge, MA, USA). Transfected cells (5×10^4^) in serum-free medium were seeded in the top chamber, and the bottom chamber was filled with medium containing 10% FBS as a chemoattractant. Chambers were incubated at 37°C in a humidified incubator containing 5% CO_2_. Twenty-four h later, cells that migrated to the underside of the membrane were fixed with 4% paraformaldehyde (Sigma Aldrich, St. Louis, MO), stained with crystal violet (Beyotime, Shanghai, China), imaged, and counted under a microscope (Leica, UK).

### Western blotting

Proteins were separated on a 12% SDS-PAGE gel and transferred onto a nitrocellulose membrane (Bio-Rad, Hercules, USA). The membrane was blocked with 5% non-fat milk and incubated with anti-β-catenin, anti-MMP2, anti-c-Myc, anti-Cyclin D1 (Santa Cruz, CA) or anti-β-actin antibodies (Sigma, CA, USA). After extensive washing, goat anti-mouse secondary antibody (Pierce, IL, USA) was added to the system. Proteins were detected using enhanced chemiluminescence reagents (Pierce).

### Statistical analysis

All statistical analyses were carried out using the SPSS 18.0 statistical software package. Continuous variables were expressed as means ± SEM. Differences between groups were calculated using Student's *t* test. *P*<0.05 (two-tailed) was considered statistically significant.
